# Reevaluation of a classic phylogeographic barrier: new techniques reveal the influence of microgeographic climate variation on population divergence

**DOI:** 10.1002/ece3.576

**Published:** 2013-04-25

**Authors:** J Angel Soto-Centeno, Lisa N Barrow, Julie M Allen, David L Reed

**Affiliations:** 1 Department of Biology, University of FloridaGainesville, Florida, 32611; 2 Florida Museum of Natural History, University of FloridaGainesville, Florida, 32611; 3 Department of Biological Science, Florida State UniversityTallahassee, Florida, 32306

**Keywords:** Apalachicola river, Florida, geographic barrier, *Geomys pinetis*, phylogeography

## Abstract

We evaluated the mtDNA divergence and relationships within *Geomys pinetis* to assess the status of formerly recognized *Geomys* taxa. Additionally, we integrated new hypothesis-based tests in ecological niche models (ENM) to provide greater insight into causes for divergence and potential barriers to gene flow in Southeastern United States (Alabama, Florida, and Georgia). Our DNA sequence dataset confirmed and strongly supported two distinct lineages within *G. pinetis* occurring east and west of the ARD. Divergence date estimates showed that eastern and western lineages diverged about 1.37 Ma (1.9 Ma–830 ka). Predicted distributions from ENMs were consistent with molecular data and defined each population east and west of the ARD with little overlap. Niche identity and background similarity tests were statistically significant suggesting that ENMs from eastern and western lineages are not identical or more similar than expected based on random localities drawn from the environmental background. ENMs also support the hypothesis that the ARD represents a ribbon of unsuitable climate between more suitable areas where these populations are distributed. The estimated age of divergence between eastern and western lineages of *G. pinetis* suggests that the divergence was driven by climatic conditions during Pleistocene glacial–interglacial cycles. The ARD at the contact zone of eastern and western lineages of *G. pinetis* forms a significant barrier promoting microgeographic isolation that helps maintain ecological and genetic divergence.

## Introduction

Almost a decade before the word phylogeography was coined, Avise et al. (1979) used molecular techniques to identify population subdivisions east and west of the Apalachicola River discontinuity (ARD) in the southeastern pocket gopher (Geomyidae: *Geomys pinetis*). Years after, a wealth of molecular data has shown that some taxa exhibit reciprocal monophyly across the ARD (Avise 2000; and references therein), whereas others appear to be unaffected (e.g., *Diadophis punctatus*, Fontanella et al. 2008; *Lampropeltis getula*, Pyron and Burbrink 2009; and *Nerodia erythrogaster*, Makowsky et al. 2010). To understand why some species show divergences across the ARD and others do not, different types of information are necessary including the ecological and evolutionary history of the taxa in question.

The Apalachicola Embayment, described by Soltis et al. (2006) as the ARD, became inundated in the Pliocene (ca. 5.6–2.6 Ma; Randazzo 1997). Glacial–interglacial cycles and the formation and rearrangement of rivers and embayments intensified barriers to dispersal and affected the phylogeographic patterns of many organisms in the southeastern United States (Soltis et al. 2006; Burbrink et al. 2008). A major geographic barrier like the ARD might influence historical processes such as population subdivision, long distance dispersal, or range expansion. For example, the pocket gophers studied by Avise et al. (1979) were shown to have a genetic break consistent with the ARD.

**Figure 1:**
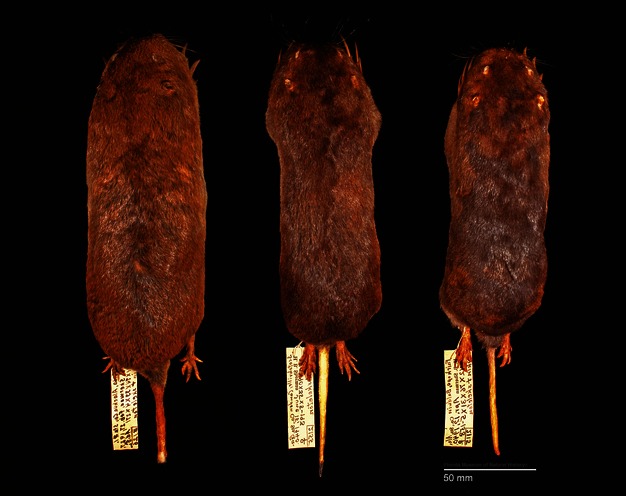
Specimens of *Geomys pinetis*. Left to Right: *G. p. pinetis* from Alachua, FL (UF10321); *G. p. colonus* from Camden, GA (UF12350); *G. p. mobilensis* from Baldwin, AL (UF12310). Photograph by J. Angel Soto-Centeno.

Pocket gophers are fossorial rodents whose distribution extends from North to Central America. *Geomys pinetis* is basal to all other species within the family Geomyidae and is the only pocket gopher in the southeastern United States, occurring throughout southern Georgia, southern Alabama, and the northern two thirds of Florida (Fig. 1; Hall 1981). Within its distribution, five subspecies are currently recognized based on morphological evidence: *G. p. austrinus*, *G. p. floridanus*, *G. p. goffi*, and *G. p. pinetis* distributed east of the ARD, and *G. p. mobilensis* distributed west of the ARD (Pembleton and Williams 1978). It is unknown whether the designated subspecies correspond to genetically distinct entities. For example, Avise et al. (1979) used allozyme data to sample many individuals from throughout the range of *G. pinetis*, but focused only on the marked genetic difference between the populations east and west of the ARD. More recently, Sudman et al. (2006) and Chambers et al. (2009) found results consistent with Avise et al. (1979; i.e., large sequence divergence east and west of the ARD), but only evaluated three individuals and did not address differences among populations or subspecies. The sampling in our study is more comprehensive; therefore, we can evaluate the mtDNA divergence and relationships within *G. pinetis* and assess the status of formerly recognized *Geomys* taxa using the cytochrome *b* gene (cyt *b*). In order to better understand the east/west split at the ARD, however, new approaches linking evolutionary history with ecological data are needed.

Ecological niche modeling (ENM) has been used for assessing the predicted geographic distributions of organisms in biogeographic, conservation, and ecological contexts (Elith and Leathwick 2009; Elith et al. 2011). ENM methods combine species occurrence and environmental data to make a predictive model of the environmental conditions that are suitable for a species to persist. Although ENMs are commonly performed on the whole distribution of species, recent studies have shown that ENM on populations can provide important information about population level differentiation because it allows the model to pick up subtle environmental differences reflecting local variation (Gonzalez et al. 2011). Therefore, modeling the distribution of populations can be useful to study the role of geographic barriers on patterns of population divergence (Graham et al. 2004; Glor and Warren 2011).

We sequenced mtDNA and developed ENMs to study the phylogeography of *G. pinetis*. Specifically, we estimated the age of the proposed divergence between eastern and western populations of *G. pinetis*, examined the status of formerly described taxa (i.e., *G. colonus, G. fontanelus,* and *G. p. goffi*; Hall 1981), tested the similarity of climatic niches for eastern and western populations, and assessed whether the ARD is a barrier separating eastern and western populations of *G. pinetis*. This approach integrates multiple sources of evidence to investigate the effect of a classic geographic barrier on populations. The combination of these methods can help us develop hypotheses to be used in future studies with other codistributed plants and animals and to assess common patterns of population subdivision across geographic barriers.

## Materials and Methods

### Samples

We amplified mitochondrial cyt *b* sequences from 58 specimens (Table S1). Tissues analyzed included 51 historical specimens collected during 1938–1983 and seven specimens collected during 2007–2008. All specimens were collected throughout the distribution of *G. pinetis* and curated in the Florida Museum of Natural History Mammal Collection. The use of historical specimens allowed us to sample along the ARD more densely and sample populations that are now extirpated. Collection of modern specimens followed guidelines approved by the American Society of Mammalogists (Sikes et al. 2011).

### Mitochondrial DNA from modern specimens

We isolated DNA from liver tissue following the protocols of the DNeasy Blood and Tissue Kit (QIAGEN, Valencia, CA). Over 1100 bp of the mitochondrial cyt *b* gene were amplified by polymerase chain reaction (PCR) using the primers H15915 (Irwin et al. 1991) and MVZ05 (Smith and Patton 1993). PCR reactions consisted of 2.5X 5 PRIME MasterMix (5 PRIME, Inc., Gaithersburg, MD), 0.4 μmol/L each primer, and 1 μL template DNA in a total volume of 25 μL. The temperature profile followed an initial denaturation at 94°C for 5 min, then 35 cycles of denaturation at 94°C for 45 sec, annealing at 45°C for 1 min, and extension at 65°C for 1 min, and a final extension at 65°C for 10 min. Double-stranded sequences were obtained using internal primers 530F and 574R (Table S2), which were designed using Primer3 v0.4.0 (Rozen and Skaletsky 1999).

### Mitochondrial DNA from historical specimens

Skin samples of ∼3 mm by 3 mm squares were cut from the abdomen of each specimen and stored in 95% EtOH at −20°C in a laboratory designated for work with ancient DNA. Prior to extraction, samples were transferred to 250 μL of phosphate-buffered saline 1× solution, washed once, and soaked for 24 h at 4°C. Extractions were performed using the QIAamp DNA Micro Kit (Qiagen, Valencia, CA) following the manufacturer's recommendations. We used negative controls on extractions and PCRs to test for contamination from modern DNA. All tubes, racks, tips, and pipettes were kept under a UV hood that was turned on for 12 min to avoid contamination before setting up each reaction. Similarly, countertops and any pipettes and tip boxes used outside of the hood were wiped down with a 10% bleach solution before and after setting up each reaction.

A double-stranded portion of the mitochondrial cyt *b* gene was amplified in small, overlapping fragments ranging in size from 150–250 bp. Primers used for both PCR and sequencing reactions were designed using Primer3 (Rozen and Skaletsky, 1999; Table S2). PCR reactions consisted of high fidelity 2X Accuzyme Mix (Bioline USA, Inc., Boston, MA), 5 U of *Taq* DNA Polymerase (Bioline USA, Inc., Boston, MA), 0.2 μmol/L each primer, and 2 μL template DNA in a total volume of 50 μL. PCR temperature profile was the same as the one used for modern tissue samples. However, for some samples the annealing temperature was reduced to 41°C and number of cycles increased to 40 to improve PCR success.

PCR products from modern and historical specimens were purified using ExoSAP-IT (USB Corporation, Cleveland, OH). Sequencing reactions were performed at the University of Florida DNA Sequencing Core Laboratory following ABI Prism BigDye Terminator cycle sequencing protocols (Applied Biosystems, Foster City, CA). Sequences were edited in sequencher v4.2.2 (Gene Codes Corporation, Ann Arbor, MI) and aligned by eye using macclade v4.06 (Sinauer Associates, Inc., Sunderland, MA; data matrix and trees in TreeBase: http://purl.org/phylo/treebase/phylows/study/TB2:S14015). Redundant sequences were removed from the dataset prior to phylogenetic analysis.

### Phylogenetic inference

Analyses were performed on a 901 bp portion of cyt *b*. Out-group sequences (*Pappogeomys bulleri* and *Geomys breviceps breviceps*) were obtained from GenBank (accession numbers L11900.1 and AY393939.1, respectively). Phylogenetic analyses were performed using maximum-likelihood and Bayesian approaches. We estimated an appropriate model of nucleotide evolution using paup* v4.0b10 (Swofford 2003) to generate the best maximum-likelihood tree. The chosen model that best fit the data corresponded to a GTR + I + G with 1-transition rate and 2-transversion rates (rclass = a b a c b c). A heuristic maximum-likelihood search was performed using TBR branch swapping, 1 random addition sequence replicate for the first two iterations, and 10 random addition sequence replicates for the final iteration. Nodal support was estimated by conducting 100 maximum-likelihood bootstrap replicates and a 50% majority rule consensus tree was generated.

Bayesian phylogenetic analysis was performed using mrBayes v3.1.2 (Ronquist and Huelsenbeck 2003) and the GTR + I + G model. The analysis consisted of two 10,000,000-generation runs with four Markov chains, sampled every 2000 generations. The log-likelihood scores were plotted against the number of generations to assess stationarity and the first 300 trees were discarded as burn-in. A 50% majority rule consensus tree was generated to calculate posterior probabilities.

### Divergence dating

We obtained additional cyt *b* sequences from GenBank for members of the family Geomyidae to estimate divergence time within *G. pinetis*. These included three additional genera of the tribe Geomyini (*Orthogeomys, Cratogeomys,* and *Pappogeomys*), additional species of the genus *Geomys*, and three species of the tribe Thomomyini (*Thomomys monticola*, *T. talpoides*, and *T. mazama*), which were used as out-groups in the following analyses. We tested for saturation in our dataset because mitochondrial third positions have shown heterogeneity in base composition when *Thomomys* and genera of the Geomyini are included (Spradling et al. 2004). Total pairwise sequence divergence was calculated and compared with third codon position pairwise sequence divergence calculated in paup* v4.0b10 (Swofford 2003). Pairwise divergence of the third codon position plotted against the total pairwise divergences did not asymptote suggesting they have not become saturated (linear fit *r*^2^ = 0.997; data not shown). Therefore, all of the data were used in the following analyses.

In a revision of the Geomyidae using morphometric data from both extant and extinct species, Russell (1968) suggested that a rapid radiation near the beginning of the Blancan North American Land Mammal Age (ca. 5 Ma) resulted in the diversification of at least four lineages that led to the modern genera of the Geomyini. Therefore, we used this 5 Ma date as a fossil calibration with a narrow range of 0.5 Ma around it (Spradling et al. 2004). A maximum-likelihood analysis was performed with the methods described above to generate a best tree for the Geomyidae. Divergence dates were calculated with the program r8s v1.71 (Sanderson 2003) using Penalized Likelihood (PL) and Non-Parametric Rate Smoothing (NPRS), and with the program beast v1.7.2 (Drummond and Rambaut 2007) using an HKY + I + G model yule process. The beast analysis was run for 30 × 10^6^ generations sampling every 2000 generations. All posterior distributions were verified for stationarity in the program tracer v1.5 (Rambaut and Drummond, 2009) after a burn-in of 10%. We used a prior of 5 Ma (SD = 5 × 10^5^) for the r8s and beast analyses. This prior was also applied in beast using a uniform distribution.

### Ecological niche modeling

**Figure 2:**
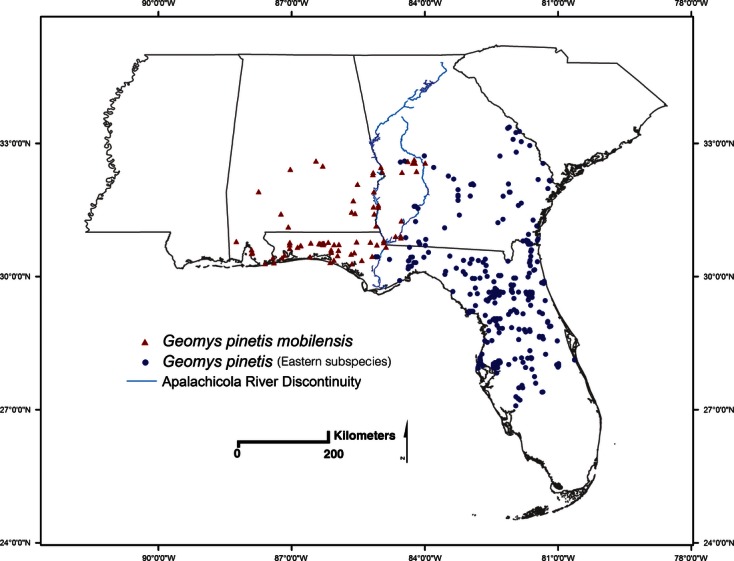
Study region in the southeastern United States including point localities of eastern *Geomys pinetis* (blue circles) and *G. p. mobilensis* (red triangles).

We used presence-only data under a maximum entropy approach to explore predicted climatic niche suitability for *G. pinetis*. Locality records with latitude/longitude coordinates for all *G. pinetis* were obtained via MaNIS (http://manisnet.org) and include newly collected records from this study. We plotted distributions of *G. p. mobilensis* and eastern *G. pinetis* separately in arcGIS v9.2 (ESRI, Redlands, CA) to assess the quality of spatial data. We corrected erroneously georeferenced localities following guidelines provided by MaNIS and confirmed the identification of specimens along the ARD. The final dataset included 369 records of the eastern *G. pinetis* and 81 records of *G. p. mobilensis* (Fig. 2). This dataset includes the most complete locality information for eastern *G. pinetis* and *G. p. mobilensis* spanning the narrow heterogeneous environmental conditions of the southeastern U.S.

We used climatic data from 19 WorldClim variables at a 30-sec (ca. 1 km^2^) spatial resolution (Hijmans et al. 2005) and soil data from USDA Soil Data Mart (http://soildatamart.nrcs.usda.gov). Both datasets were clipped to a regional extent of southeastern United States using arcGIS v9.2. The WorldClim climate variables represent annual trends, seasonality, and extremes of temperature and precipitation. Soil data represent the most detailed soil type and chemical properties dataset for the region. We performed a Pearson Correlation in R v2.15.0 to remove correlated climatic variables (correlation coefficient >0.8; Peterson 2011). The resulting dataset included eight climatic variables (i.e., Temperature: mean diurnal range, bio 2; max of warmest month, bio 5; mean of wettest quarter, bio 8; and mean of driest quarter, bio 9. Precipitation: annual, bio 12; wettest month, bio 13; seasonality, bio 15; and wettest quarter, bio 16). ENMs were generated using maxent v3.3.3 (Phillips et al. 2006; Phillips and Dudík 2007).

We were interested in determining whether climatic regions occupied by *G. pinetis* east and west of the ARD differed. Therefore, we used the genetic boundaries (i.e., eastern and western clades) of *G. pinetis* to partition the species into two populations and examined them separately using ENMs (Gonzalez et al. 2011). The use of all locality records from eastern *G. pinetis* and *G. p. mobilensis* without subsampling can result in overly fit distributions when modeling in such a narrow geographic space. However, our goal was not to describe all parts of environmental space that are outside each population's true ecological niche. The inclusion of all locality records allowed us to define the subtle environmental differences in geographic space that define the distribution of each population east and west of the ARD. We extracted climate information for our species data and for two random background climate datasets of 500 points for *G. p. mobilensis* and 800 points for eastern *G. pinetis* to sample the background climate and to ensure replicability of the ENMs. We used the auto-features in maxent and allowed the algorithm to reach convergence in each of the replicates excluding any duplicate localities from the dataset. ENMs consisted of 100 bootstrap replicates using the respective climatic background of each population, and projected to the southeastern United States. Bootstrap replicates consisted of sampling with replacement of each training dataset (i.e., 75% of presence localities for both eastern *G. pinetis* and *G. p. mobilensis*). Model performance was evaluated using the area under receiver operating characteristic curve (AUC), where values >0.7 are characteristic of good model performance (Swets 1988). Some authors suggest that evaluating model performance using AUC is not appropriate in some cases (e.g., when using presence-only data; Lobo et al. 2008). Therefore, we applied a lowest presence threshold of 95% (LPT95%) to the average model from maxent logistic output to generate final predictions and assess model performance. In this assessment, we used a model sensitivity analysis through a binomial one-tailed test in R v2.15.0 to estimate if the true positive fraction of the models is significantly greater than that generated by a random model. We chose an LPT95% because it is a relaxed threshold for which predicted distributions result in at least 95% of all occurrences falling into suitable habitat (i.e., a 5% omission rate). The relaxed stringency of LPT95% also allowed us to make better estimates of the overlap between distributions. Finally, we used the range overlap test in ENMtools to estimate the amount of overlap between distributions of eastern *G. pinetis* versus *G. p. mobilensis* (Warren et al. 2010).

### Assessing the ARD as a geographic barrier

To learn if a significant geographic barrier separates populations of *G. pinetis* east and west of the ARD, we first tested the similarity of the ENMs of each population by calculating niche similarity indices *I* and Schoener's *D* using ENMtools v1.3 (Warren et al. 2008, 2010). Specifically, these metrics compare whether ENMs generated from *G. pinetis* east and west of the ARD are identical (niche identity test) or if ENMs obtained from the two allopatrically distributed populations are more different than expected given the environmental differences between the regions in which they occur (background similarity test). To estimate niche identity, ENMtools generates a null distribution of overlap scores obtained from a shared distribution between populations (Warren et al. 2010). For the background test, a null distribution is generated for the ENM difference between one population and a random sample of the background climate available to the other population (Warren et al. 2010). In this case, ENMs of each allopatric population show environmental divergence if the empirical values obtained for each population are significantly different from values obtained from the random samples of the background. Results from the identity and background tests are compared to the empirical *I* and Schoener's *D* values using one-tailed and two-tailed *t*-tests for the niche identity and background identity tests, respectively.

Additionally, we tested whether the ARD represents an abrupt environmental barrier or an area of unsuitable climate between two suitable regions. To assess if the ARD represents an abrupt barrier, we used a linear randomization analysis implemented in the “blob” range-breaking test of ENMtools (Fig. S1; Glor and Warren 2011). The linear “blobs” are generated by pooling locality records of *G. pinetis* east and west of the ARD, selecting a single point at random, and linearly expanding from that point to partition the dataset to match the number of locality records for eastern *G. pinetis* and *G. p. mobilensis*. To assess if the ARD represents an area of unsuitable climate separating areas of higher suitability, we used the random ribbon range-breaking test (Glor and Warren 2011). The width of the “ribbon” of unsuitable climate was estimated using ArcGIS v9.2 based on the width of the contact zone of eastern *G. pinetis* and *G. p. mobilensis* along the ARD (i.e., 70.8 km = 0.63 decimal degrees, Fig. S1). During the ribbon range-breaking test, all locality records of *G. pinetis* east and west of the ARD are pooled, then random ribbons of the specified width are generated to partition the dataset. We generated 100 random range-break replicates to generate null distributions of *I* and Schoener's *D* values for both of these tests, which were compared to the empirical values using a one-tailed *t*-test.

## Results

### Phylogenetic inference and divergence dating

**Figure 3:**
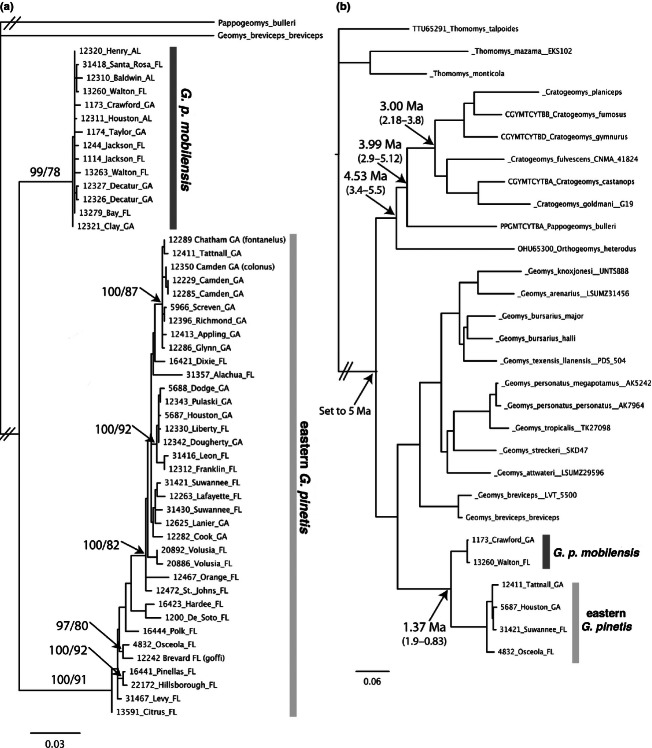
Best Maximum-Likelihood phylogram of *Geomys pinetis* (a) and best Maximum-Likelihood phylogram with divergence date estimates (b) based on cyt *b* gene sequences. (a) Phylogenetic reconstruction reveals two well-supported east and west clades in *Geomys*. Nodal values represent Bayesian posterior probabilities and maximum-likelihood bootstrap support. (b) Calibration point for divergence of Geomyidae was set at 5 Ma following Spradling et al. (2004). Numbers on nodes represent estimates of divergence from beast with 95% HPD intervals. Individual sequences downloaded from GenBank are identified with their respective accession numbers.

Eight out of 58 individuals analyzed resulted in redundant sequences and were removed from phylogenetic analyses. A total of 50 individuals of *G. pinetis* representing 43 counties in Florida, Georgia, and Alabama were studied along with two out-groups (*P. bulleri* and *G. b. breviceps*). Maximum-likelihood and Bayesian analyses recovered trees with similar topologies, thus we present the best maximum-likelihood tree (Fig. 3a).

Within *G. pinetis* there were two strongly supported monophyletic clades separated geographically by the ARD (Fig. 3a). Average uncorrected pairwise distances and K2P-corrected pairwise distances (Kimura 1980) between the eastern and western samples were 7.88% and 8.50%, respectively (Table S3). There was little differentiation within *G. p. mobilensis* (uncorrected *P* = 0.44%, K2P = 0.44%). Within the eastern *G. pinetis*, additional clades were recovered by both analyses but support values were lower and the relationships among them were unresolved (Fig. 3a). The average uncorrected pairwise difference within the eastern *G. pinetis* was 1.99% whereas the K2P-corrected distance for the eastern *G. pinetis* was 2.04%.

In general, the groups within the eastern *G. pinetis* clade occur in geographically contiguous populations that are somewhat inconsistent with formerly defined subspecies distributions (see Pembleton and Williams 1978). The individuals representing the putative subspecies *G. p. goffi* as well as the previously recognized *G. colonus* and *G. fontanelus* do not appear to be genetically distinct from surrounding populations of *G. pinetis* for cyt *b* with average genetic distances <1.0% for both uncorrected *P* and K2P distances.

Dates from the PL and NPRS method in the program r8s estimated the divergence between eastern *G. pinetis* and *G. p. mobilensis* at 1.57 Ma. This date was similar to the 1.37 Ma date estimated using beast and fell well within the 95% highest posterior density (HPD, 1.9 Ma–830 ka, Fig. 3b).

### Ecological niche modeling

**Figure 4:**
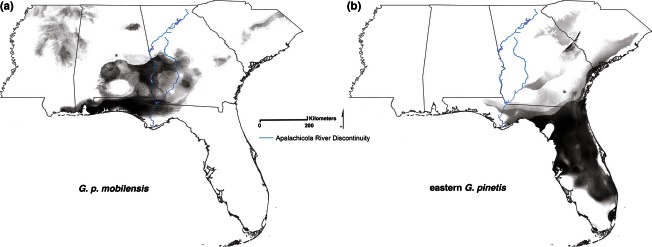
Ecological niche models generated for *Geomys p. mobilensis* (a) and eastern *G. pinetis* (b) using maxent. Levels of shading represent continuous logistic probability of occurrence based on climatic suitability. Grayscale represents decreasing suitability from highly suitable (black) to unsuitable (white). The blue lines represent the Apalachicola, Flint, and Chattahoochee Rivers, which together make up the ARD.

ENMs developed with soil data performed slightly worse than those developed using climate data alone (*G. p. mobilensis* AUC = 0.882; eastern *G. pinetis* AUC = 0.759). Therefore, we show only distribution models based on climate. These models performed well for both *G. p. mobilensis* and eastern *G. pinetis* with average AUC values of 0.909 (SD ± 0.007) and 0.773 (SD ± 0.004), respectively. Binomial sensitivity tests for each prediction were significant and confirmed model accuracy (*P* < 0.01). Predicted distributions correspond well with expectations based on molecular data and were able to define each population, with *G. p. mobilensis* primarily occurring to the west of the ARD and the remaining subspecies to the east of the ARD (Fig. 4). The highest climatic suitability for *G. p. mobilensis* occurs in areas west of the ARD with the most suitable niche located in the Florida panhandle (Fig. 4a). In contrast, the highest climatic suitability for eastern *G. pinetis* subspecies concentrates east of the ARD in peninsular Florida, with the most suitable niche located in north central Florida and the Tampa areas (Fig. 4b). The most striking feature of the distribution models is that only about 5% of the predicted distributions overlap along the ARD (Fig. 4). Climatic niche of *G. p. mobilensis* quickly declines in suitability as it extends east of the ARD and climatic niche of eastern *G. pinetis* stops almost precisely at the ARD. Furthermore, *G. p. mobilensis* models generated using background climate only from west of the ARD and projected onto the entire southeastern U.S. show low suitability to unsuitable climate to the east and into the Florida peninsula (Fig. 4a). Similar results are obtained when modeling the distribution of eastern *G. pinetis* using the same approach (Fig. 4b).

In all comparisons, we obtained similar trends from *I* and Schoener's *D* metrics; therefore, we focus our discussion on *I* because it reveals more variation than Schoener's *D* (Glor and Warren 2011). For the niche identity and background similarity tests, we obtained *I* and Schoener's *D* values greater than our empirical values (Fig. S2). This suggests that ENMs from *G. p. mobilensis* and eastern *G. pinetis* are not identical (identity test, *P* < 0.01) nor are their ENMs more similar to each other than expected by chance based on random sampling of their available backgrounds (*G. p. mobilensis* vs. eastern *G. pinetis* background, *P* < 0.01; eastern *G. pinetis* vs. *G. p. mobilensis* background, *P* < 0.01). Linear (i.e., blob) range breaking reveals that the difference in ENMs for *G. p. mobilensis* and eastern *G. pinetis* is not significantly different than that obtained by random geographic partitions (*P* = 0.672). Therefore, we reject the hypothesis that the ARD acts as an abrupt climate barrier separating populations of *G. p. mobilensis* and eastern *G. pinetis*. In contrast, ENMs developed for *G. p. mobilensis* and eastern *G. pinetis* support the hypothesis that the ARD represents a ribbon of unsuitable climate between more suitable areas where these populations are distributed (*G. p. mobilensis* vs. ARD, *P* = 0.017; eastern *G. pinetis* vs. ARD, *P* < 0.01). This result is also confirmed by the lower suitability scores obtained along the ARD in both ENMs (Fig. 4). Furthermore, the ribbon range-break analysis also confirms the dissimilarity in climatic habitat in the flanking regions to the east and west of the ARD (*G. p. mobilensis* vs. eastern *G. pinetis*, *P* < 0.01).

## Discussion

Thirty years after the seminal work of Avise et al. (1979), we confirm the presence of two very divergent lineages that span the ARD using an independent DNA sequence dataset in *G. pinetis*. Our results also reveal little additional divergence within populations of eastern *G. pinetis*, and no difference to the formerly recognized *G. colonus, G. fontanelus,* and *G. p. goffi* (Pembleton and Williams 1978; Hall 1981). Furthermore, we estimated divergence between the *Geomys* lineages to be 1.37 Ma. This suggests that the divergence was driven by climatic conditions during Pleistocene glacial–interglacial cycles. The size and hydrology of the Florida peninsula changed drastically throughout the Pleistocene glaciations, resulting in glacial refugia that created phylogeographic breaks for multiple taxa (Soltis et al. 2006). The flood plains of the Apalachicola, Chatahoochee, and Flint Rivers (i.e., the ARD) are characterized by low elevations that have been inundated during Pleistocene interglacials. Pocket gophers have a very specialized lifestyle, disperse poorly, and are restricted to specific habitat types (Pembleton and Williams 1978). On a physiological study of fossorial mammals, McNab (1966) showed that the *G. pinetis* distribution in Florida closely matched the distribution of soils having low water-holding capacity. These sandy soils are also characterized by having high porosity that allows adequate gas diffusion to support a fossorial mammal lifestyle within a closed burrow (McNab 1966). Therefore, areas that have frequent changes in soil water-holding capacity due to inundation or increase in the level of the water table will prevent gophers from building viable burrows. Fluctuating sea levels during Pleistocene glacial–interglacial cycles likely changed soil characteristics and created wide water barriers partitioning populations of *G. p. mobilensis* and eastern *G. pinetis* along the ARD and promoting diversification over time.

Ecological divergence and adaptation to different habitats can influence the geographic distribution of species and can minimize connectivity among populations (Sobel et al. 2010). By generating ENMs at the population level, we were able to model the distributions of *G. p. mobilensis* and eastern *G. pinetis* with fine resolution and sensitivity to local adaptation. Niche conservatism is the propensity of closely related taxa to maintain characteristics of their fundamental niche (Peterson et al. 1999; Wiens and Graham 2005; Peterson 2011). Previous research revealed that some species show conservatism of ecological niches across moderate evolutionary time scales (Peterson et al. 1999). We observed a general trend of environmental divergence corresponding with genetic divergence between populations of *G. p. mobilensis* and eastern *G. pinetis* across the ARD (Figs. 4). Furthermore, results of ENMs suggest an important role of ecologically maintained divergence in which the suitable climatic niche of *G. p. mobilensis* differs significantly from that of eastern *G. pinetis* and vice versa. Niche identity and background similarity tests reveal that *G. p. mobilensis* and eastern *G. pinetis* distributions are not identical and that ENMs of these lineages are not more similar than expected by chance based on random sampling of their available background. This contrasts with the niche conservatism hypothesis because differences in climatic niche observed over the ARD in the southeastern pocket gopher show a change in climatic niche preferences on a moderate timescale and a small spatial scale. A similar scenario has been observed in high elevation species, such as frogs in the genus *Eleutherodactylus*, where altitudinal differences in distributions drives the separation of species into different climatic niches (Lynch and Duellman 1997). We suggest that climatic niche differences and the low vagility of the southeastern pocket gopher contribute to strong divergence across a short environmental gradient.

Geographic barriers play an important role in biodiversity and speciation because they directly affect the distribution of organisms and the probability of gene flow. Isolation created by geographic barriers promotes the segregation of populations, which in turn can be exposed to different ecological conditions that lead to evolutionary divergence (Mayr 1947). The ARD has long been one of the major barriers influencing the phylogeography of many species distributed in the southeastern United States (Soltis et al. 2006; and citations therein). In this study, we tested the significance of the ARD as a barrier influencing the phylogeography of *G. pinetis*. Our results strongly suggest that the ARD is a ribbon of unsuitable habitat at the contact zone of eastern *G. pinetis* and *G. p. mobilensis* rather than an abrupt barrier separating these populations. This hypothesis is further supported by the lower suitability scores obtained in ENMs of eastern *G. pinetis* and *G. p. mobilensis* within the ribbon of unsuitable habitat (Fig. 2A). Also, we observed low levels of false positives by the ENMs from each lineage modeling into the range of the other. Researchers have long identified the importance of geographic barriers as well as ecological barriers in the isolation of races and allopatric species (Stebbins 1950). Ecological barriers can influence adaptation to different environmental conditions (e.g., climatic in the case of *G. pinetis*) and potentially affect the encounter rates between populations, which in turn promote divergence. Some individuals of *G. pinetis* successfully crossed the rivers in the ARD. These dispersal events could have occurred during glacial periods because the lower sea and water table levels allowed for drier soil conditions where gophers can burrow. However, the marked zone of unsuitable climate in the ARD likely precluded long distance dispersal and contact between these individuals. Our models show that the ARD is a significant biogeographic barrier promoting microgeographic isolation that helps maintain ecological and genetic divergence of eastern *G. pinetis* and *G. p. mobilensis*.

## Conclusions

Biogeographic barriers are known to contribute to genetic divergence in many organisms by exposing populations to different ecological conditions. The role of the ARD as a biogeographic barrier for populations has been considered important for many plants and animals, including the southeastern pocket gopher (*Geomys pinetis*). Our analysis explores the significance of the ARD in maintaining *G. pinetis* population differences. This approach provided an ecological explanation for a longstanding question proposed by Avise et al. (1979) about maintenance of genetic divergence between *G. pinetis* populations east and west of this biogeographic barrier. Making general conclusions about the importance of the ARD as a biogeographic barrier requires a comparative approach evaluating many taxa in the way we have done here. Nevertheless, the observed presence of different climatic conditions maintaining microgeographic isolation of populations and the significance of the ARD as a barrier to gene flow between eastern *G. pinetis* and *G. p. mobilensis* help us understand and generate testable hypotheses about divergences in other species found in the southeastern United States. This integrative framework of ENMs and genetics has a lot of potential for deciphering patterns of diversification and to understand comparative evolutionary histories at local or regional geographic scales.

## Biosketch

**J. Angel Soto-Centeno** is a doctoral candidate at the Department of Biology and Florida Museum of Natural History, University of Florida. His research focuses on phylogeography and understanding the effects of climate change on mammalian population diversification by integrating ecological niche models, fossils, and genetics.

## Supporting Information

**Figure S1.** Examples of linear blob (A) and ribbon test (C) for our empirical data and two random linear blobs and ribbons respectively (B and D). The width of the ribbon was estimated based on the easternmost locality for *Geomys p. mobilensis* and the westernmost locality for eastern *G. pinetis*. The width of the ribbon was calculated using ArcGIS (70.8 km = 0.63 decimal degrees) and was maintained at the same width for all 100 randomized iterations. In linear blobs, gray points represent localities comprising the size of the smallest dataset (*G. p. mobilensis*) and black points represent localities comprising the size of the largest dataset (eastern *G. pinetis*). In the ribbon test, gray points represent localities within the ribbon and black points represent pooled localities from areas flanking the ribbon.

**Figure S2.** Results from niche identity tests showing the empirical values for *I* and Schoener's *D* (black and gray arrows). Density plot indicates the distribution of values for *I* and Schoener's *D* (black and gray bars) after 100 iterations. Values of *I* and Schoener's *D* are significantly lower than expected based on the null hypothesis of niche identity (i.e., niches of *Geomys p. mobilensis* and eastern *G. pinetis* are not identical; *P* < 0.001).

**Table S1.** Specimens used for phylogenetic analyses including GenBank numbers. Asterisks represent specimens with redundant sequences that were removed from phylogenetic analyses.

**Table S2.** List of new sequencing and PCR primers designed to amplify 901 bp of the mitochondrial cytochrome *b* gene in *Geomys pinetis*.

**Table S3.** Average pairwise genetic distances between selected taxa calculated using uncorrected p and Kimura 2-parameter model of evolution (K2P, Kimura 1980).
