# Supplementary material for: Reevaluation of a classic phylogeographic barrier: new techniques reveal the influence of microgeographic climate variation on population divergence
Source: Ecol Evol. 2013 Apr 25;3(6):1603–13. doi: 10.1002/ece3.576 (PMC3686195; doi:10.1002/ece3.576)
Supplement: Supplementary file 1 [file ece30003-1603-SD1.docx]

Fig. S1. Examples of linear blob (a) and ribbon test (c) for our empirical data and two random linear blobs and ribbons respectively (b and d). The width of the ribbon was estimated based on the easternmost locality for *G. p. mobilensis* and the westernmost locality for eastern *G. pinetis.* The width of the ribbon was calculated using ArcGIS (70.8km = 0.63 decimal degrees) and was maintained at the same width for all 100 randomized iterations. In linear blobs, gray points represent localities comprising the size of the smallest dataset (*G. p. mobilensis*) and black points represent localities comprising the size of the largest dataset (eastern *G. pinetis*). In the ribbon test, gray points represent localities within the ribbon and black points represent pooled localities from areas flanking the ribbon.

Fig. S2. Results from niche identity tests showing the empirical values for *I* and Schoener’s *D* (black and gray arrows). Density plot indicates the distribution of values for *I* and Schoener’s *D* (black and gray bars) after 100 iterations. Values of *I* and Schoener’s *D* are significantly lower than expected based on the null hypothesis of niche identity (i.e. niches of *G. p. mobilensis* and eastern *G. pinetis* are not identical; *P* < 0.001).

Table S1. Specimens used for phylogenetic analyses including GenBank numbers. Asterisks represent specimens with redundant sequences that were removed from phylogenetic analyses.

| Species | Catalogue No. | Locality | Latitude | Longitude | Year Collected | Genbank Accession No. |
| --- | --- | --- | --- | --- | --- | --- |
| *G. pinetis* | UF1114 | Jackson, FL | 30.7874 | -84.9439 | 1955 | HQ170567 |
| *G. pinetis* | UF1173 | Crawford, GA | 32.5597 | -83.9931 | 1957 | HQ170563 |
| *G. pinetis* | UF1174 | Taylor, GA | 32.6353 | -84.2383 | 1957 | HQ170575 |
| *G. pinetis* | UF1200 | De Soto, FL | 27.2156 | -81.8586 | 1957 | HQ170594 |
| *G. pinetis* | UF1244 | Jackson, FL | 30.6664 | -84.8767 | 1957 | HQ170578 |
| *G. pinetis* | UF4832 | Osceola, FL | 28.0068 | -80.9881 | 1958 | HQ170597 |
| *G. pinetis* | UF5687 | Houston, GA | 32.458 | -83.8005 | 1957 | HQ170566 |
| *G. pinetis* | UF5688 | Dodge, GA | 32.1974 | -83.3493 | 1957 | HQ170560 |
| *G. pinetis* | UF5965* | Screven, GA | 32.9534 | -81.6369 | 1960 | HQ170600* |
| *G. pinetis* | UF5966 | Screven, GA | 32.9534 | -81.6369 | 1960 | HQ170568 |
| *G. pinetis* | UF12229 | Camden, GA | 30.7997 | -81.6765 | 1938 | HQ170564 |
| *G. pinetis* | UF12242 | Brevard, FL | 28.1289 | -80.6306 | 1938 | HQ170606 |
| *G. pinetis* | UF12263 | Lafayette, FL | 29.9589 | -82.9283 | 1939 | HQ170569 |
| *G. pinetis* | UF12282 | Cook, GA | 31.0969 | -83.5564 | 1939 | HQ170587 |
| *G. pinetis* | UF12285 | Camden, GA | 30.7779 | -81.69 | 1939 | HQ170604 |
| *G. pinetis* | UF12286 | Glynn, GA | 31.0364 | -81.4522 | 1939 | HQ170570 |
| *G. pinetis* | UF12289 | Chatham, GA | 32.1654 | -81.1969 | 1939 | HQ170572 |
| *G. pinetis* | UF12291* | Chatham, GA | 32.1654 | -81.1969 | 1939 | HQ170603* |
| *G. pinetis* | UF12292* | Chatham, GA | 32.1654 | -81.1969 | 1939 | HQ170590* |
| *G. pinetis* | UF12310 | Baldwin, Al | 30.5228 | -87.9033 | 1940 | HQ170571 |
| *G. pinetis* | UF12311 | Houston, Al | 31.1411 | -85.0967 | 1940 | HQ170565 |
| *G. pinetis* | UF12312 | Franklin, FL | 29.9092 | -84.5689 | 1940 | HQ170598 |
| *G. pinetis* | UF12319* | Clay, GA | 31.6089 | -85.0472 | 1940 | HQ170602* |
| *G. pinetis* | UF12320 | Henry, Al | 31.57 | -85.1593 | 1940 | HQ170559 |

Table S1. Continued.

| Species | Catalogue No. | Locality | Latitude | Longitude | Year Collected | Genbank Accession No. |
| --- | --- | --- | --- | --- | --- | --- |
| *G. pinetis* | UF12321 | Clay, GA | 31.5639 | -85.0472 | 1940 | HQ170608 |
| *G. pinetis* | UF12326 | Decatur, GA | 30.9036 | -84.6601 | 1940 | HQ170593 |
| *G. pinetis* | UF12327 | Decatur, GA | 30.9036 | -84.5417 | 1940 | HQ170592 |
| *G. pinetis* | UF12330 | Liberty, FL | 30.3864 | -84.7981 | 1940 | HQ170573 |
| *G. pinetis* | UF12342 | Dougherty, GA | 31.5348 | -84.1558 | 1940 | HQ170582 |
| *G. pinetis* | UF12343 | Pulaski, GA | 32.2647 | -83.4722 | 1940 | HQ170584 |
| *G. pinetis* | UF12350 | Camden, GA | 30.7689 | -81.6175 | 1940 | HQ170576 |
| *G. pinetis* | UF12396 | Richmond, GA | 33.3682 | -82.098 | 1941 | HQ170585 |
| *G. pinetis* | UF12411 | Tattnall, GA | 31.9014 | -82.0982 | 1941 | HQ170580 |
| *G. pinetis* | UF12413 | Appling, GA | 31.2575 | -81.285 | 1941 | HQ170561 |
| *G. pinetis* | UF12467 | Orange, FL | 28.5997 | -81.3394 | 1944 | HQ170605 |
| *G. pinetis* | UF12472 | St. Johns, FL | 30.0758 | -81.6475 | 1945 | HQ170607 |
| *G. pinetis* | UF12625 | Lanier, GA | 31.385 | -83.2231 | 1949 | HQ170562 |
| *G. pinetis* | UF13260 | Walton, FL | 30.3183 | -86.1303 | 1974 | HQ170589 |
| *G. pinetis* | UF13262* | Walton, FL | 30.3183 | -86.1303 | 1974 | HQ170609* |
| *G. pinetis* | UF13263 | Walton, FL | 30.3183 | -86.1303 | 1974 | HQ170588 |
| *G. pinetis* | UF13267* | Walton, FL | 30.3183 | -86.1303 | 1974 | HQ170601* |
| *G. pinetis* | UF13279 | Bay, FL | 30.5525 | -85.3905 | 1974 | HQ170599 |
| *G. pinetis* | UF13591 | Citrus, FL | 29.014 | -82.4212 | 1976 | HQ170581 |
| *G. pinetis* | UF16421 | Dixie, FL | 29.6011 | -82.9819 | 1980 | HQ170591 |
| *G. pinetis* | UF16423 | Hardee, FL | 27.493 | 81.8845 | 1980 | HQ170583 |
| *G. pinetis* | UF16441 | Pinellas, FL | 28.0194 | -82.7719 | 1980 | HQ170577 |
| *G. pinetis* | UF16444 | Polk, FL | 28.1524 | -81.6019 | 1980 | HQ170596 |
| *G. pinetis* | UF20886 | Volusia, FL | 28.8715 | -81.192 | 1983 | HQ170579 |

Table S1. Continued.

| Species | Catalogue No. | Locality | Latitude | Longitude | Year Collected | Genbank Accession No. |
| --- | --- | --- | --- | --- | --- | --- |
| *G. pinetis* | UF20892 | Volusia, FL | 29.1874 | -81.3241 | 1983 | HQ170574 |
| *G. pinetis* | UF22144* | Hillsborough, FL | 27.9472 | -82.4586 | 1975 | HQ170595* |
| *G. pinetis* | UF22172 | Hillsborough, FL | 27.9472 | -82.4586 | 1983 | HQ170586 |
| *G. pinetis* | UF31356* | Alachua, FL | 29.5074 | -82.1719 | 2007 | HQ170553* |
| *G. pinetis* | UF31357 | Alachua, FL | 29.5074 | -82.1719 | 2007 | HQ170552 |
| *G. pinetis* | UF31416 | Leon, FL | 30.4145 | -84.085 | 2008 | HQ170556 |
| *G. pinetis* | UF31418 | Santa Rosa, FL | 30.627 | -86.858 | 2008 | HQ170555 |
| *G. pinetis* | UF31421 | Suwannee, FL | 30.1977 | -82.4596 | 2008 | HQ170554 |
| *G. pinetis* | UF31430 | Suwannee, FL | 30.1977 | -82.5791 | 2008 | HQ170558 |
| *G. pinetis* | UF31467 | Levy, FL | 29.2448 | -82.4917 | 2008 | HQ170557 |

Table S2. List of new sequencing and PCR primers designed to amplify 901 bp of the mitochondrial cytochrome *b* gene in *G. pinetis*.

| Primer | Sequence (5' - 3') |
| --- | --- |
| 530F | GCTTCTTCGCATTCCACTTT |
| 574R | GGGCTGRGATAATAAATGGT |
| 108F | TGACCTACCAACACCACCAA |
| 353R | CCTCGTCCAATGTGGATGTAT |
| 158alF | YACATCMGATACCCTAACAGC |
| 429alR | TAACTGTRGCCCCTCARAAT |
| 308F | GAGCCTCCCTRTTTTTCATC |
| 557R | GAAAAYCCACCTCAGATYCA |
| 417alF | GGGGCYACAGTTATTYCCAA |
| 641alR | RCCACAGTCTGATGGRATTC |
| 616F | ATCGCAGCYCTAGYAATAGT |
| 863R | TCTGGYTTAATGTGTGGTGKAG |
| 793F | GACAAAYTRGGAGACCCAGA |
| 1077R | TGGYTGTCCTCCAATTCATGT |

Table S3. Average pairwise genetic distances between selected taxa calculated using uncorrected p and Kimura 2-parameter model of evolution (K2P, Kimura 1980).

| Taxa | Uncorrected p (%) | K2P (%) |
| --- | --- | --- |
| Eastern vs. Western | 7.88 | 8.50 |
| Within Western | 0.44 | 0.44 |
| Within Eastern | 1.99 | 2.04 |
| *colonus* vs. Camden | 0.11 | 0.11 |
| *colonus* vs. Group IA | 0.33 | 0.33 |
| *fontanelus* vs. Tatnall | 0.22 | 0.22 |
| *fontanelus* vs. Group IA | 0.32 | 0.32 |
| *goffi* vs. Osceola | 0.89 | 0.90 |
