# Supplementary figures and images for: Reevaluation of a classic phylogeographic barrier: new techniques reveal the influence of microgeographic climate variation on population divergence
Source: Ecol Evol. 2013 Apr 25;3(6):1603–13. doi: 10.1002/ece3.576 (PMC3686195; doi:10.1002/ece3.576)

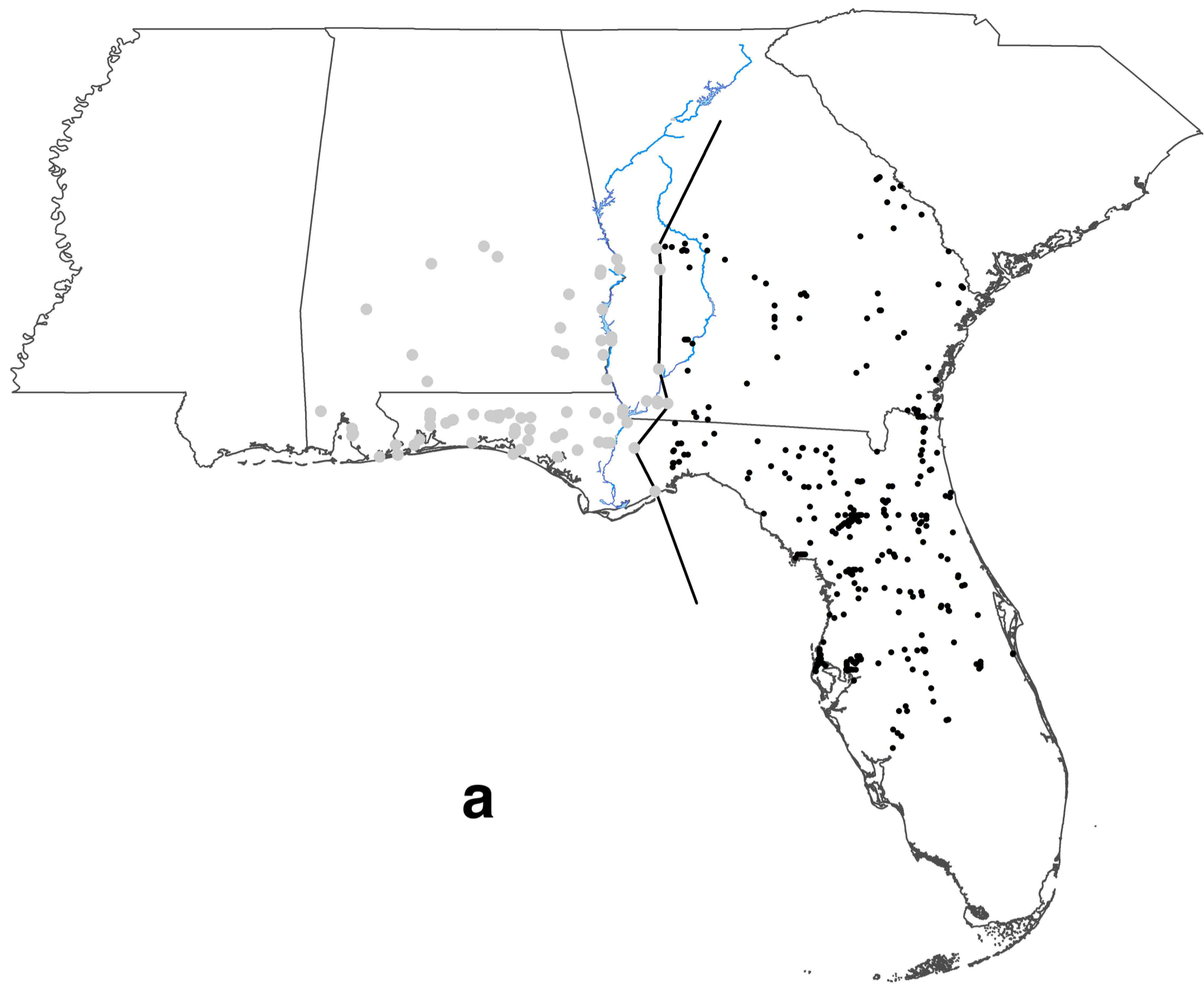

**a**

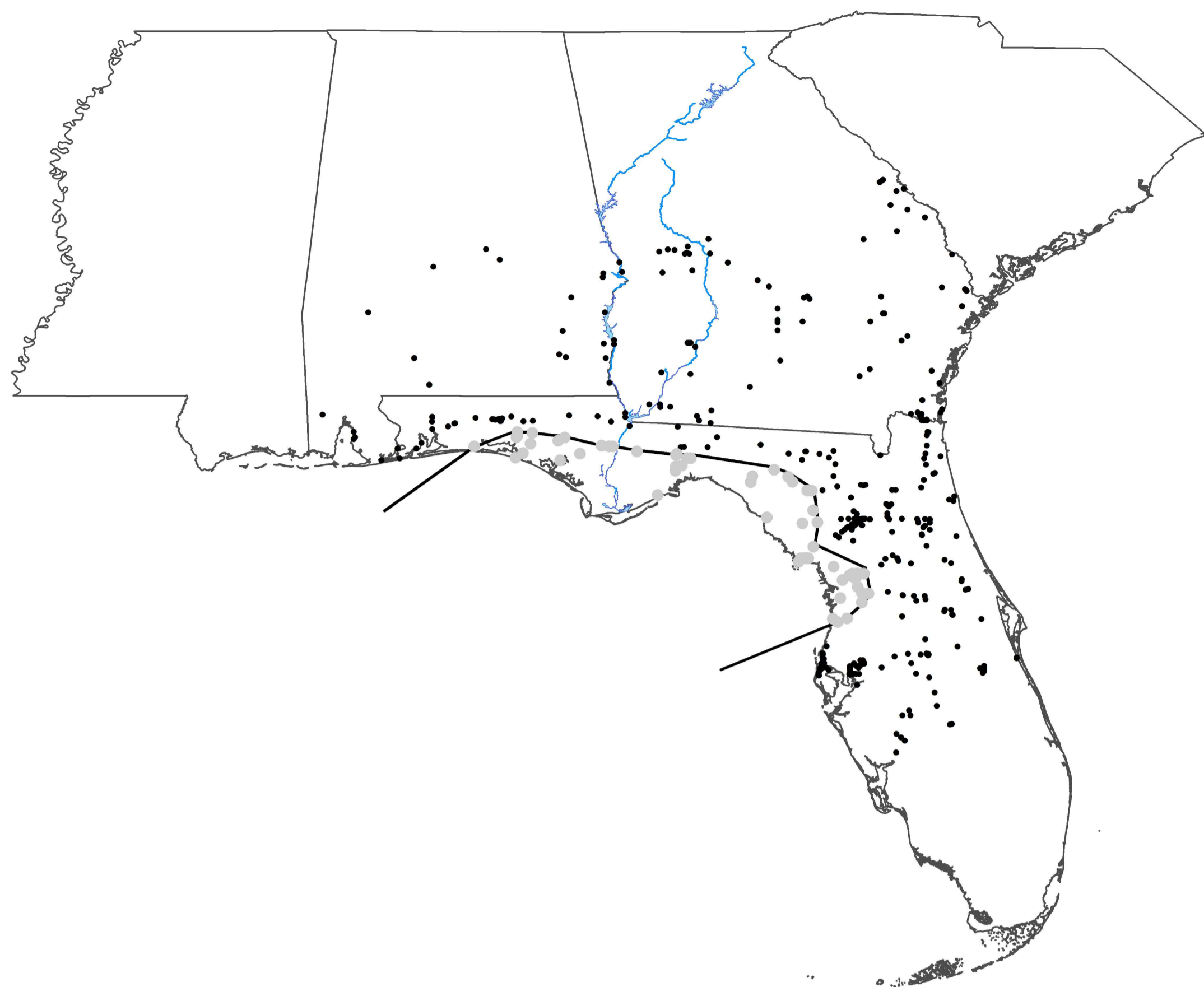

**b**

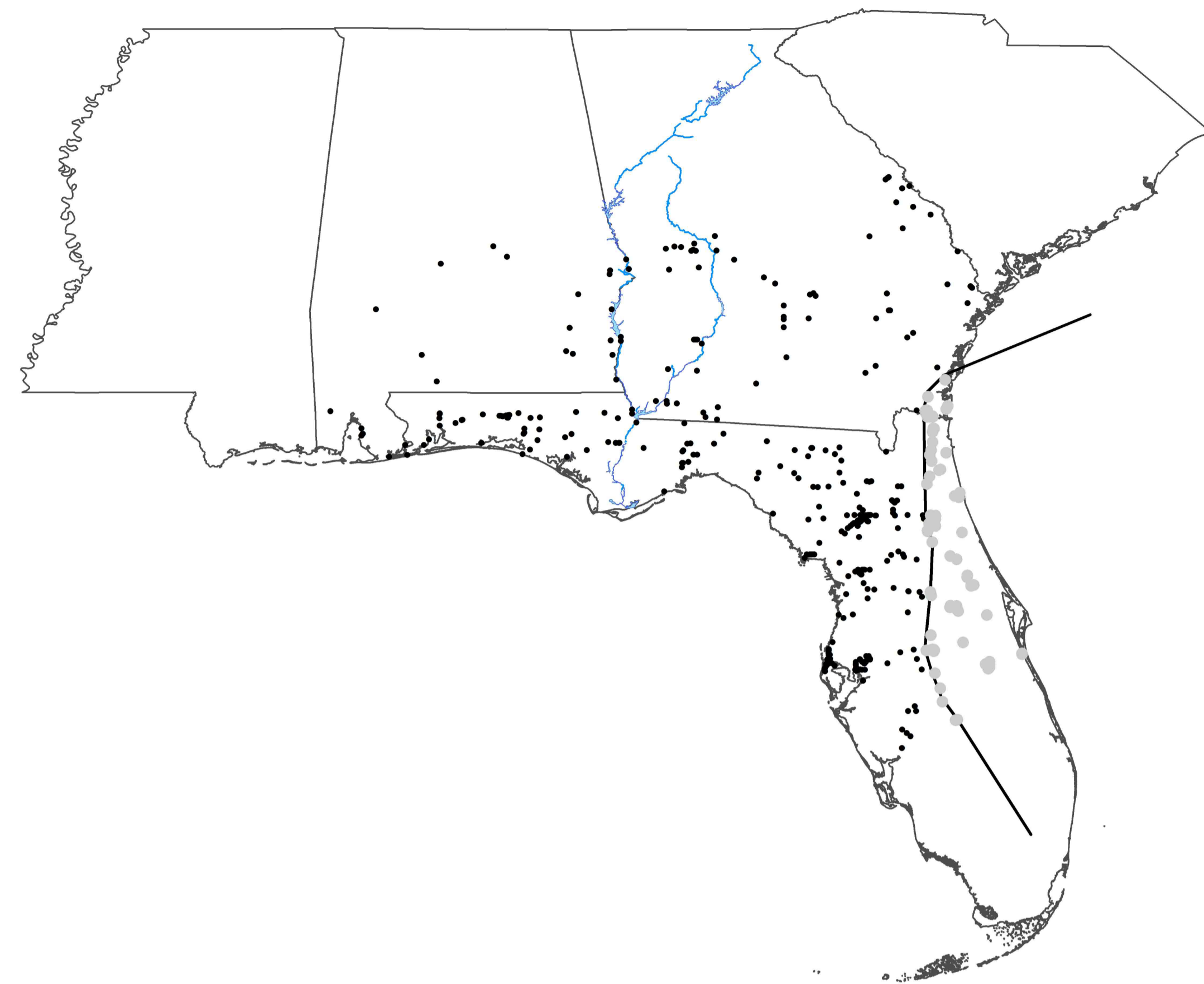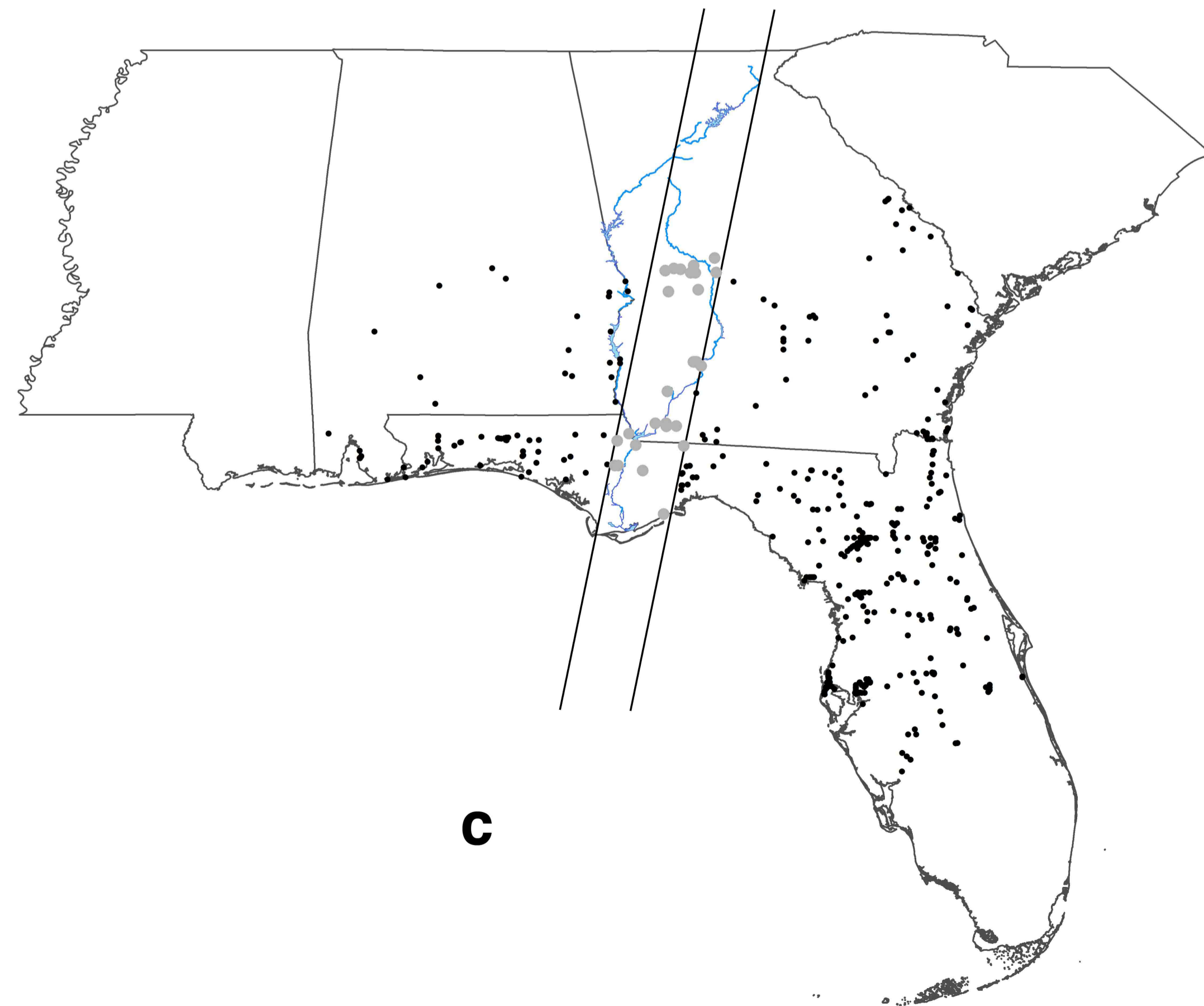

**c**

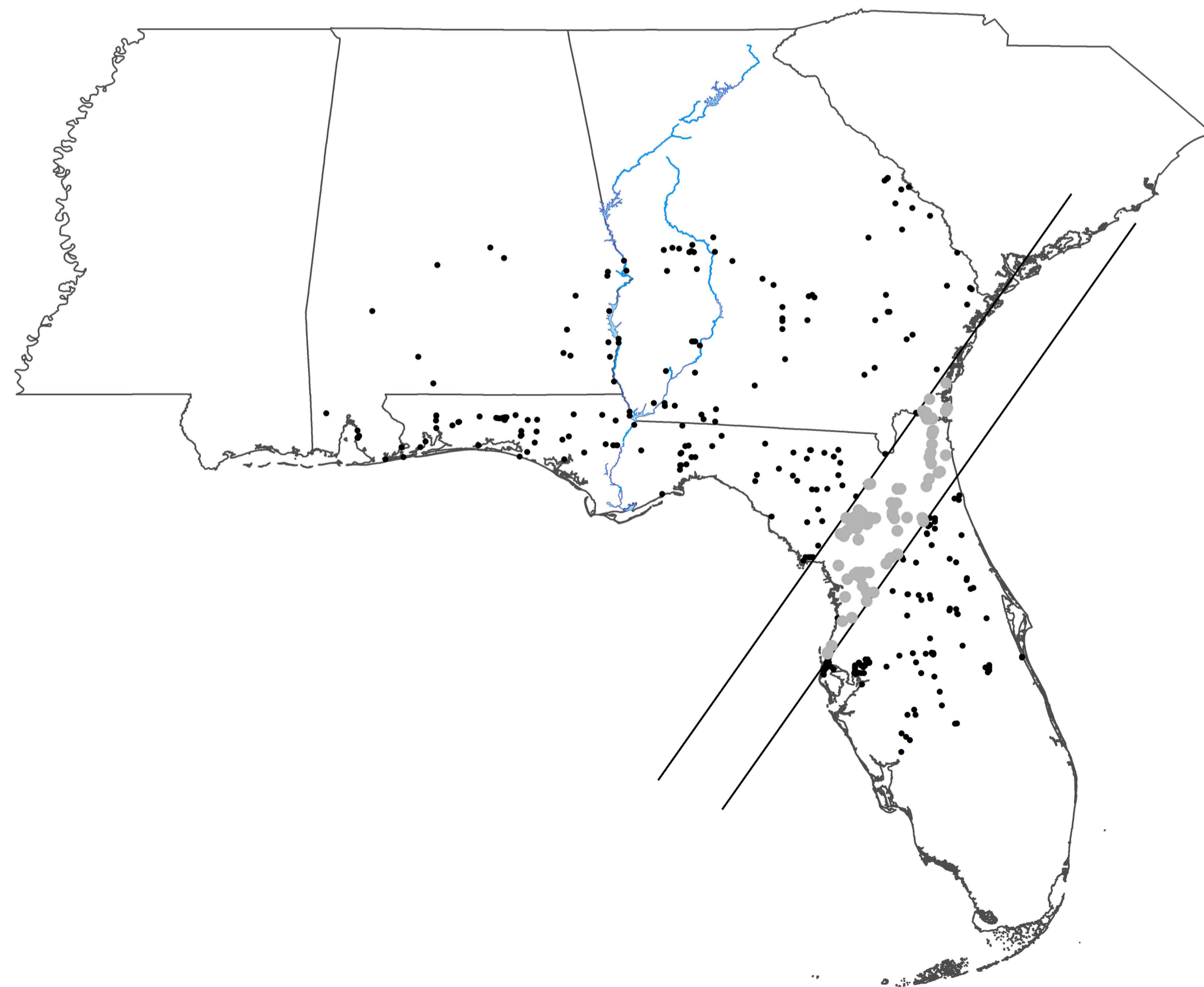

**d**

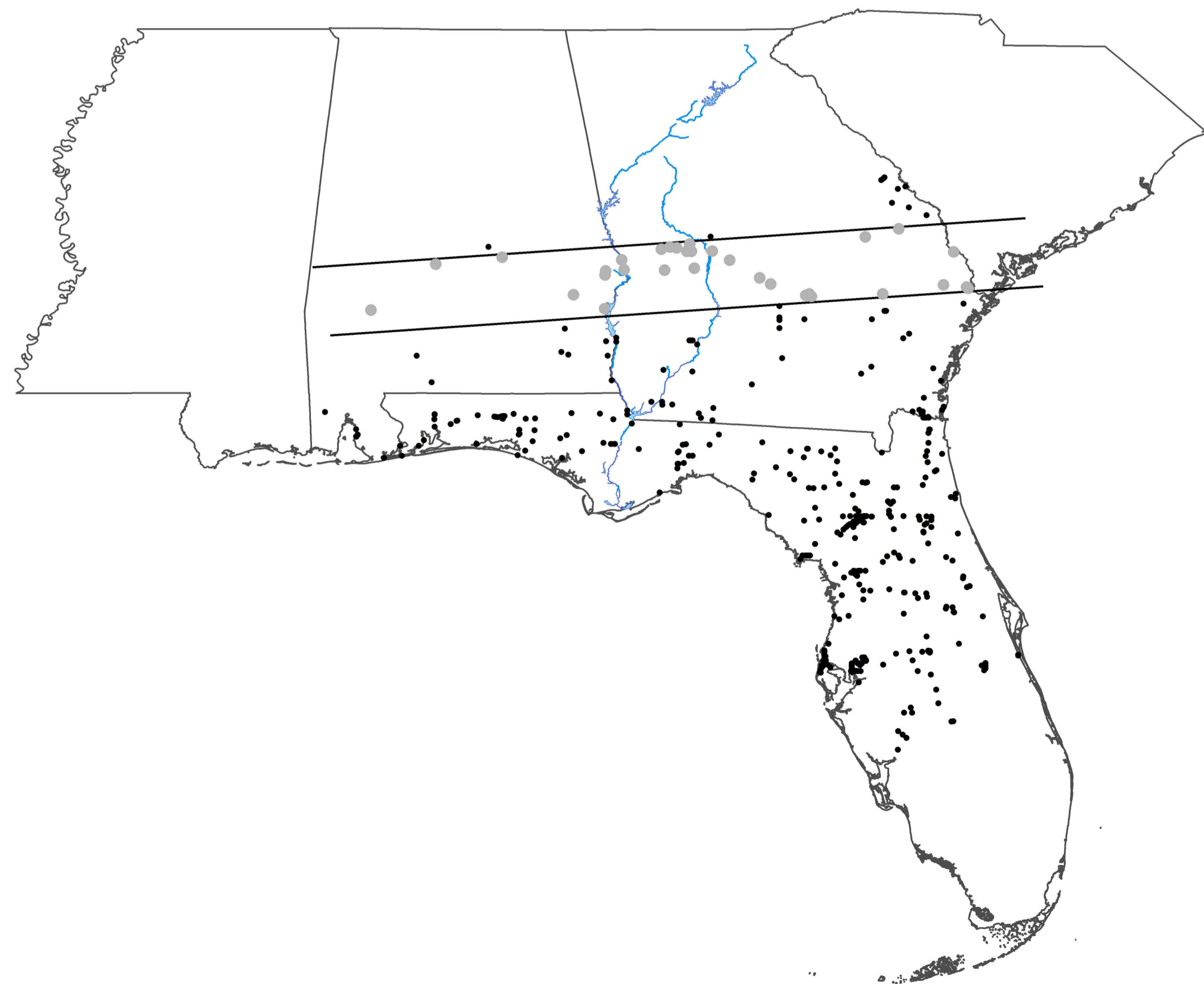

Supplement: Supplementary file 2 [file ece30003-1603-SD2.pdf]

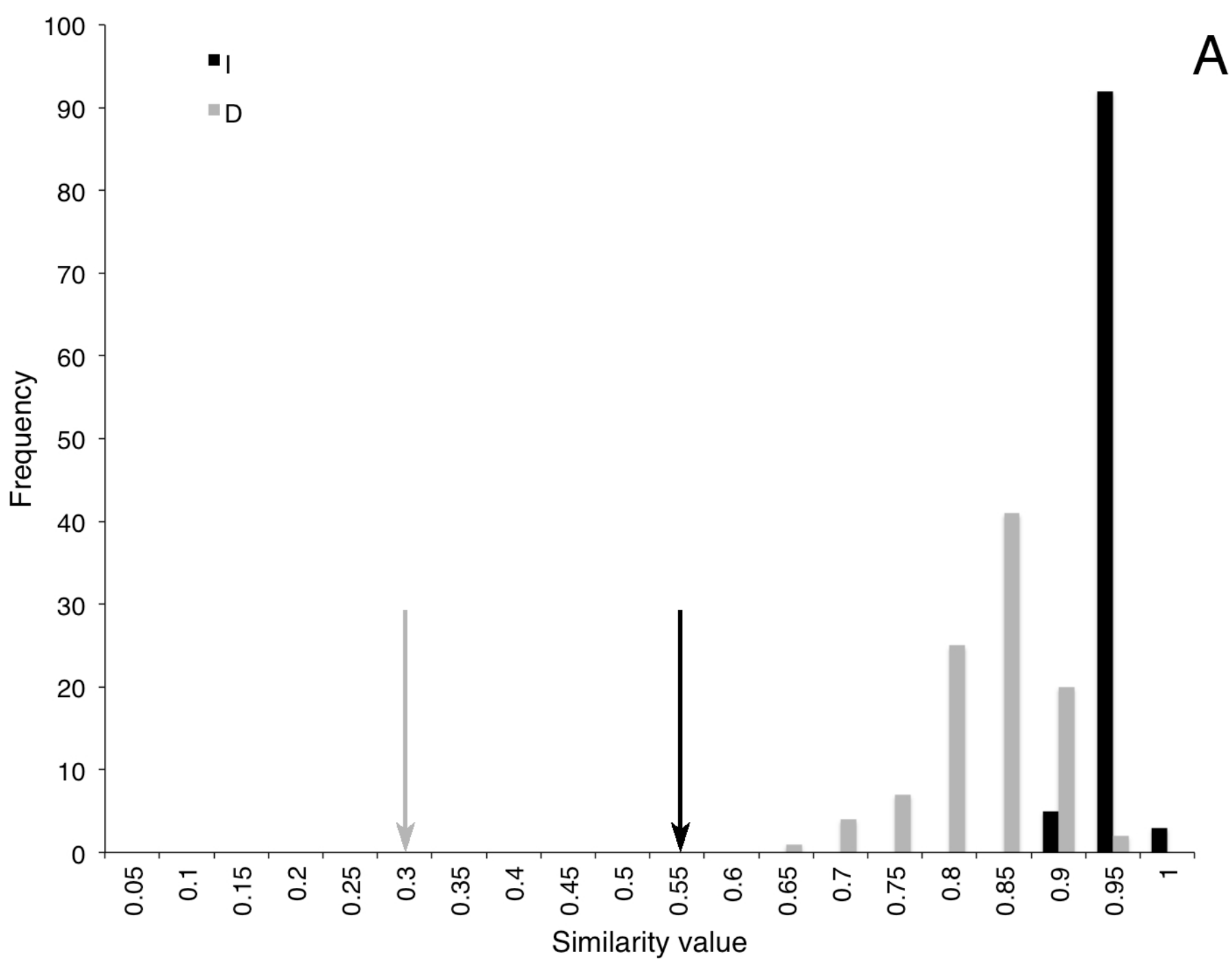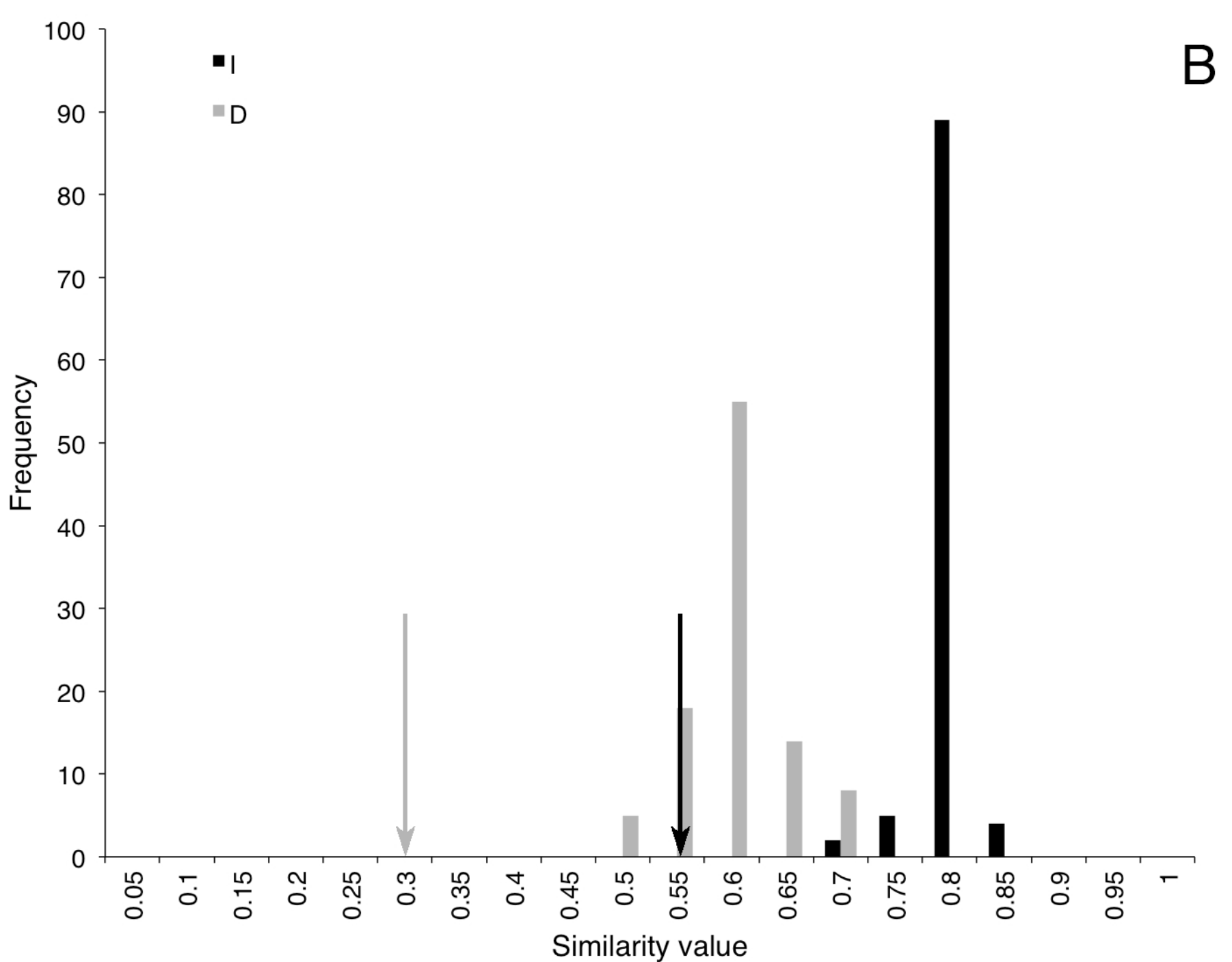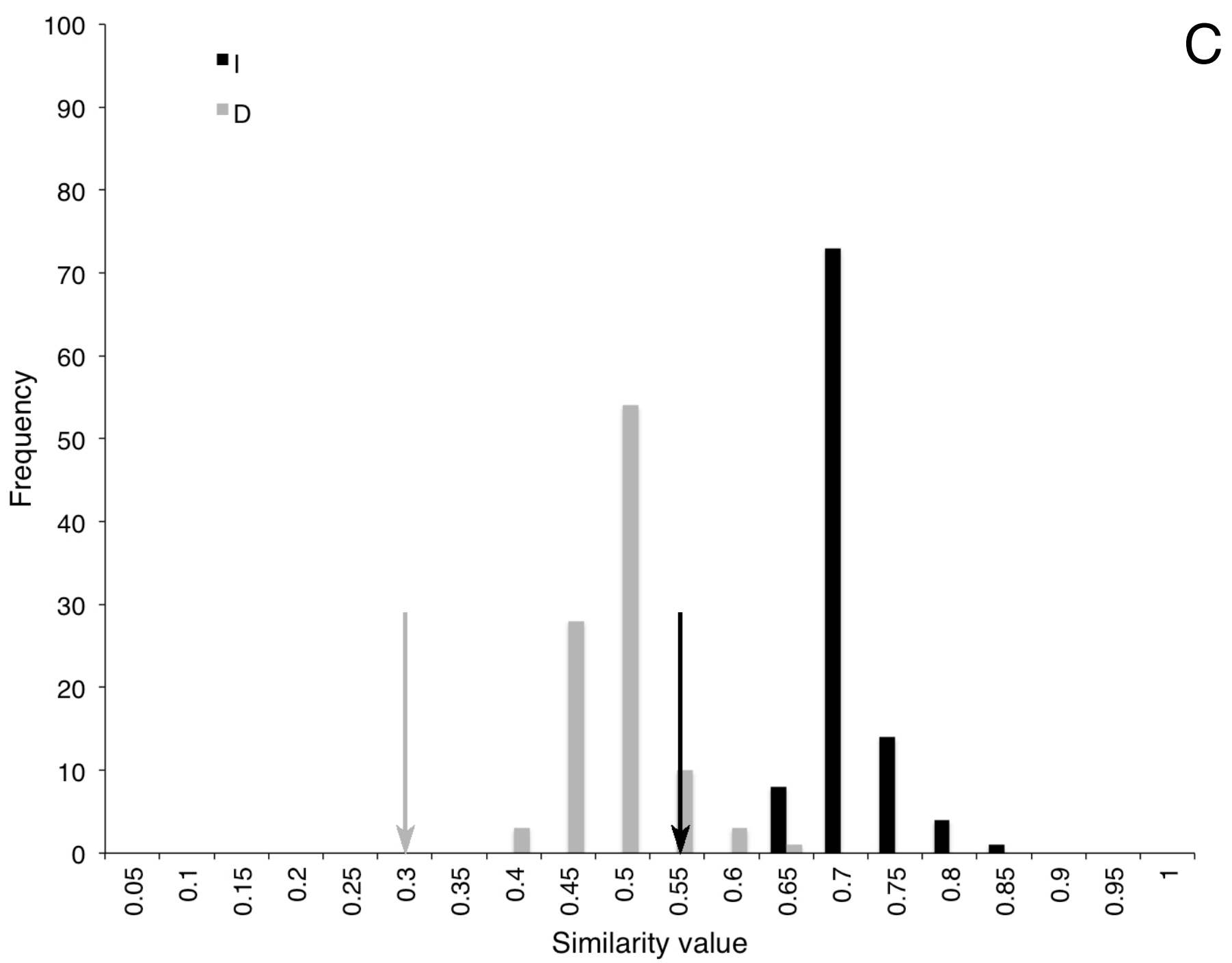

Supplement: Supplementary file 3 [file ece30003-1603-SD3.pdf]
